# Non-invasive VOCs detection to monitor the gut microbiota metabolism in-vitro

**DOI:** 10.1038/s41598-024-66303-7

**Published:** 2024-07-09

**Authors:** Andrea Dell’Olio, Josep Rubert, Vittorio Capozzi, Matteo Tonezzer, Emanuela Betta, Vincenzo Fogliano, Franco Biasioli

**Affiliations:** 1 Food Quality and Design, Wageningen University & Research, 6708 WG Wageningen, Netherlands; 2 Reserach and Innovation Centre, Fondazione Edmund Mach, 39098 San Michele All’Adige, Italy; 3 Institute of Food Production Sciences, National Research Council, 71121 Foggia, Italy; 4 Department of Chemical and Geological Sciences, University of Cagliari, 09042 Monserrato , Italy

**Keywords:** Gut microbiota, Temporal profile, Diet, Metabolomics, Gastrointestinal models, High-throughput screening

## Abstract

This work implemented a non-invasive volatile organic compounds (VOCs) monitoring approach to study how food components are metabolised by the gut microbiota in-vitro. The fermentability of a model food matrix rich in dietary fibre (oat bran), and a pure prebiotic (inulin), added to a minimal gut medium was compared by looking at global changes in the volatilome. The substrates were incubated with a stabilised human faecal inoculum over a 24-h period, and VOCs were monitored without interfering with biological processes. The fermentation was performed in nitrogen-filled vials, with controlled temperature, and tracked by automated headspace-solid-phase microextraction coupled with gas chromatography–mass spectrometry. To understand the molecular patterns over time, we applied a multivariate longitudinal statistical framework: repeated measurements—ANOVA simultaneous component analysis. The methodology was able to discriminate the studied groups by looking at VOCs temporal profiles. The volatilome showed a time-dependency that was more distinct after 12 h. Short to medium-chain fatty acids showed increased peak intensities, mainly for oat bran and for inulin, but with different kinetics. At the same time, alcohols, aldehydes, and esters showed distinct trends with discriminatory power. The proposed approach can be applied to study the intertwined pathways of gut microbiota food components interaction in-vitro.

## Introduction

The human endogenous intestinal microflora, which comprises trillions of microorganisms, is a critical component of the gut ecosystem^1,2^. Many studies investigated the composition and functionality of the microbiota in terms of metabolic potential, both using in-vitro and in-vivo systems^3–5^. Colonic batch fermentations are usually applied to evaluate the impact of dietary components on an individual’s gut microbiota in a short period of time (24–48 h)^3^. These models are useful for the initial screening of various food components and faecal donors compared to dynamic fermentation systems, which need long incubation (weeks) and restrictions in assessing multiple conditions^3,6^. Nevertheless, it remains a significant gap in understanding the dynamic, temporal interplay of faecal microbial communities when interacting with dietary components. Few studies have captured the short-term dynamics of microbial succession on dietary substrates, mainly by time-resolved genome sequencing^7,8^. As an example, De Paepe et al. revealed, by time-resolved 16S rRNA gene amplicon sequencing, how wheat bran faecal fermentation is characterised by abrupt shifts in community composition, giving rise to a succession of bacterial taxa alternately over a 72 h timespan^7^. However, tracking microbial dynamics necessitates frequent sampling, which is often hampered by lack of automation and invasive procedures. During gut fermentation, besides nonvolatile molecules, microorganisms produce a wide range of inorganic and organic volatile organic compounds (VOCs) that play important roles as signals in intra and interkingdom interactions at low concentrations and over long distances^9–12^. Microbial VOCs can be detected in a microbial culture’s gas phase and/or water phase^9–13^. These compounds have distinct physicochemical properties: they are small molecules (up to 300 Da) with up to two functional groups that can easily diffuse in air and water^10^. VOCs can travel fast and over longer distances through the liquid and gas phases^10^. Certain classes of VOCs, such as organic acids, are good indicators of gut metabolic activities, and can be considered non-invasive biomarkers in gastrointestinal health^12,14–20^. The most studied VOCs produced within the gut environment are the short-chain fatty acids (SCFAs) and in particular butanoic acid has multiple roles in the host-microbe interactions^14,21,22^. In this regard, gas chromatography coupled with mass spectrometry (GC-MS) is a well-established technique often exploited for the targeted investigation of SCFAs in colonic batch fermentation studies to test how these compounds are modulated in respect to food-gut microbiota interactions^3,23^. The analysis is often performed at few time points and for a limited range of metabolites^3,23^. During intestinal in-vitro batch fermentations, apart from SCFAs, several VOCs, belonging to different chemical classes, are released into the headspace^24^. Nevertheless, only few studies applied untargeted GC-MS analysis to monitor the multitude of VOCs extracted from the fermentation supernatants during in-vitro experiments, limiting the analysis to few snapshots over the entire fermentation and not taking into account the proper statistical frameworks to handle VOCs longitudinal datasets^24–26^. The untargeted analysis of VOCs (volatilomics) in a longitudinal way, is a novel approach within metabolomics^9,27^ which deserves attention due the possibility of streamlining the cultivation, sampling and analysis process to monitor the bacterial metabolism in a non-invasive way^9,28^. We propose headspace-solid-phase microextraction (HS-SPME), with temperature-control and automated sampling operations, coupled with GC-MS to monitor VOCs released during anaerobic in-vitro gut fermentation of dietary fibres as a model example. In this regard, SPME is a well-known non-invasive preparation methodology using a fused-silica fiber that is coated on the outside with a suitable stationary phase^29^. A small quantity of the analytes present in the sample’s headspace are extracted and concentrated directly onto the coating^29^. It has been successfully automated and applied to a wide variety of chemicals in combination with GC-MS, particularly for the extraction of volatile and semi-volatile organic compounds from environmental, biological, and dietary samples^29,30^. By continuously sampling over time colonic in-vitro fermentations by HS-SPME-GC-MS, we aim to assess the changes in VOCs when the microbiota is exposed to dietary fibres. To interpret the longitudinal data, we apply a comprehensive and advanced statistical framework suited for time-series studies such as repeated measurements: ANOVA simultaneous component analysis (RM-ASCA)^31^. In detail, we compare the VOCs time evolution when a human faecal inoculum is exposed to a complex minimal gut medium alone or supplemented with inulin (a known prebiotic) or oat bran (a complex food matrix high in dietary fibre and resistant to digestion in the upper gastrointestinal tract^32^). The key objectives of this work are: (1) to demonstrate the possibility of non-invasive, automated VOCs monitoring during in-vitro gut microbial fermentation over 24 h; (2) to distinguish the fermentation events at volatilome level by considering the longitudinal, multivariate nature of these data.

## Results

### Hierarchical clustering of longitudinal VOCs data obtained over 24 h fermentation

**Figure 1:**
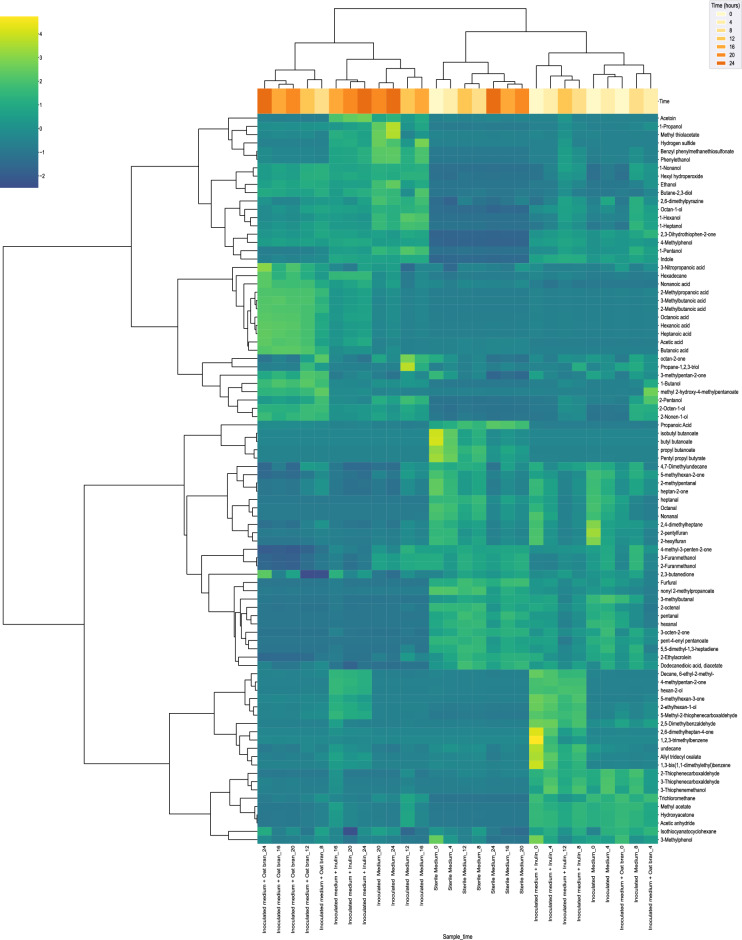
Clustermap of VOCs temporal profile across 4 sample type. The samples were monitored for 24 h every 4 h (0, 4, 8, 12, 16, 20, 24 h). The analysed samples were: inoculated medium with oat bran, inoculated medium with inulin, inoculated basal medium and sterile medium (control). The samples are listed at the bottom, and the VOCs are listed along the right-hand side. The expression levels of VOCs are represented by color scales, with yellow indicating high expression levels and blue indicating low expression levels. The dendrogram illustrates the clustering of the samples with similar VOCs profiles. The cluster map showed how the samples clustered in the early and late fermentation stages. Samples in the early fermentation stage (pale yellow) clustered together on the right-hand side of the dendrogram, while samples in the late fermentation stages clustered together on the right-hand side (dark yellow). Data are representative of three independent experiments (n = 3).

During the in-vitro anaerobic fecal batch fermentation, the emission of volatile organic compounds (VOCs) was monitored every 4 h for 24 h. This fermentation was conducted using a minimal gut medium supplemented with either oat bran or inulin as fermentable substrates. To facilitate comparison, the VOCs emitted by the basal medium alone (which contained mucin as the sole fermentable substrate) were also analyzed. Additionally, sterile medium samples (non-inoculated) were included and analyzed as controls for VOC release over time. The molecules produced by the gut microbiota over time were captured using a completely automated procedure that included cultivation, sampling, and VOC analysis. A graphical representation of the experimental approach is shown in Supplementary material (Fig. S1). The experimental setup allowed the longitudinal monitoring of 88 VOCs consistently detected across all replicates. The compounds that were not consistently detected were discarded from the analysis. The cluster map in Fig. 1 summarised the overall dataset and clustered together molecules with similar time-dependent profiles. Data indicated that samples in the same fermentation stage showed similar expression profiles. Observing the similarity cluster in the left part of the figure, it was possible to detect two large clusters that generated four sub-clusters (Clusters I–IV).

From top to bottom, the subcluster I, which was more abundant in the medium-late stages of oat bran and inulin fermentations, included mainly alcohols with multiple chain lengths and substitutions (1-Butanol, 1-Propanol, Ethanol, 2,3-Butanediol, 1-Nonanol, 2-Octen-1-ol, 2-Nonen-1-ol). This VOC cluster was slightly induced in the late fermentation stages of dietary fibres, more pronounced for the inulin samples compared to oat bran. The sub-cluster II was mainly composed of medium- and short-chain fatty acids and long-chain alcohols. The fatty acids were clearly more present in the medium-late stages of oat bran fermentation, but also detected in the final stages of inulin fermentation. The third cluster (Cluster III) was the largest in terms of the number of VOCs. In this group, there were different chemical classes including aldehydes and esters, but also ketones, furans, alkanes, and others. VOCs in this group generally showed similar peak intensities in the early stages of fermentation for all sample types and are constant in the sterile medium cluster. This was the core VOCs composition generated by the medium and did not change drastically within the first hours of fermentation for the inoculated samples. Cluster IV included different chemical classes, including thiophenes, ketones, alkanes and alcohols. All VOCs associated with this group showed higher abundances for inulin samples in the early fermentation stages. Overall, the map showed how the samples clustered in terms of metabolic profile, in the early and late stages of fermentation. As expected, the sterile medium (control samples) clustered together showing no temporal evolution. The VOCs profile of inoculated samples at early-stage fermentation was similar to the sterile medium, but a clear evolution in time was evident at late stage. The same trend was visible in Supplementary material (Fig. S2) where the sterile media samples grouped together in the score plot of a principal component analysis (PCA).

### Time-dependent multivariate analysis of VOCs by RM-ASCA

**Figure 2:**
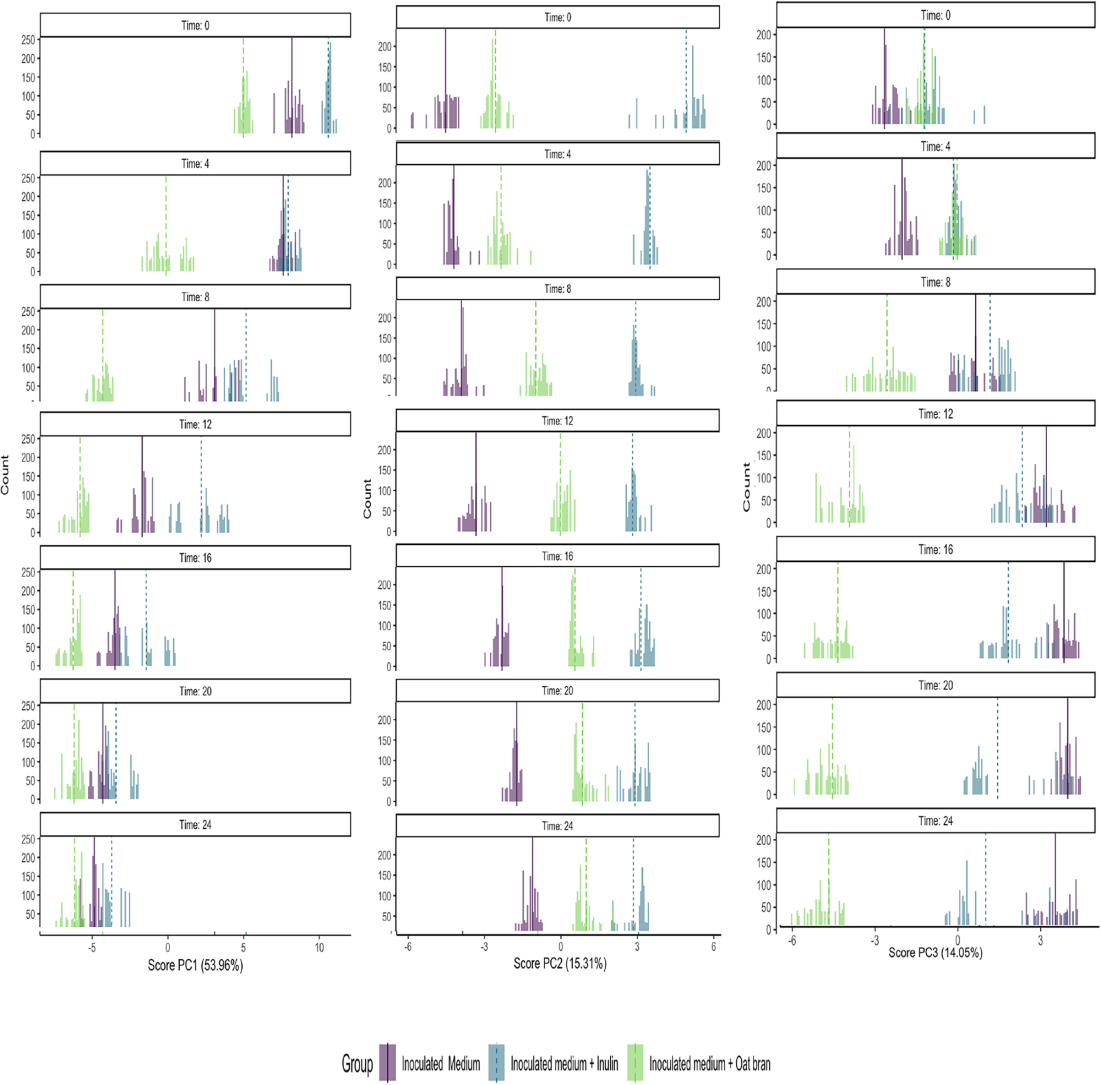
Distribution of scores for PC1 , PC2 and PC3 as histograms. The three groups were basal medium + inoculum + inulin (blue), basal medium + inoculum + oat bran (green), and basal medium + inoculum (violet). The visualisation capture the key events in VOCs profile throughout the fermentation process. Data were representative of three independent experiments. The distribution of scores is obtained by jack-knife validation n = 1000.

**Figure 3:**
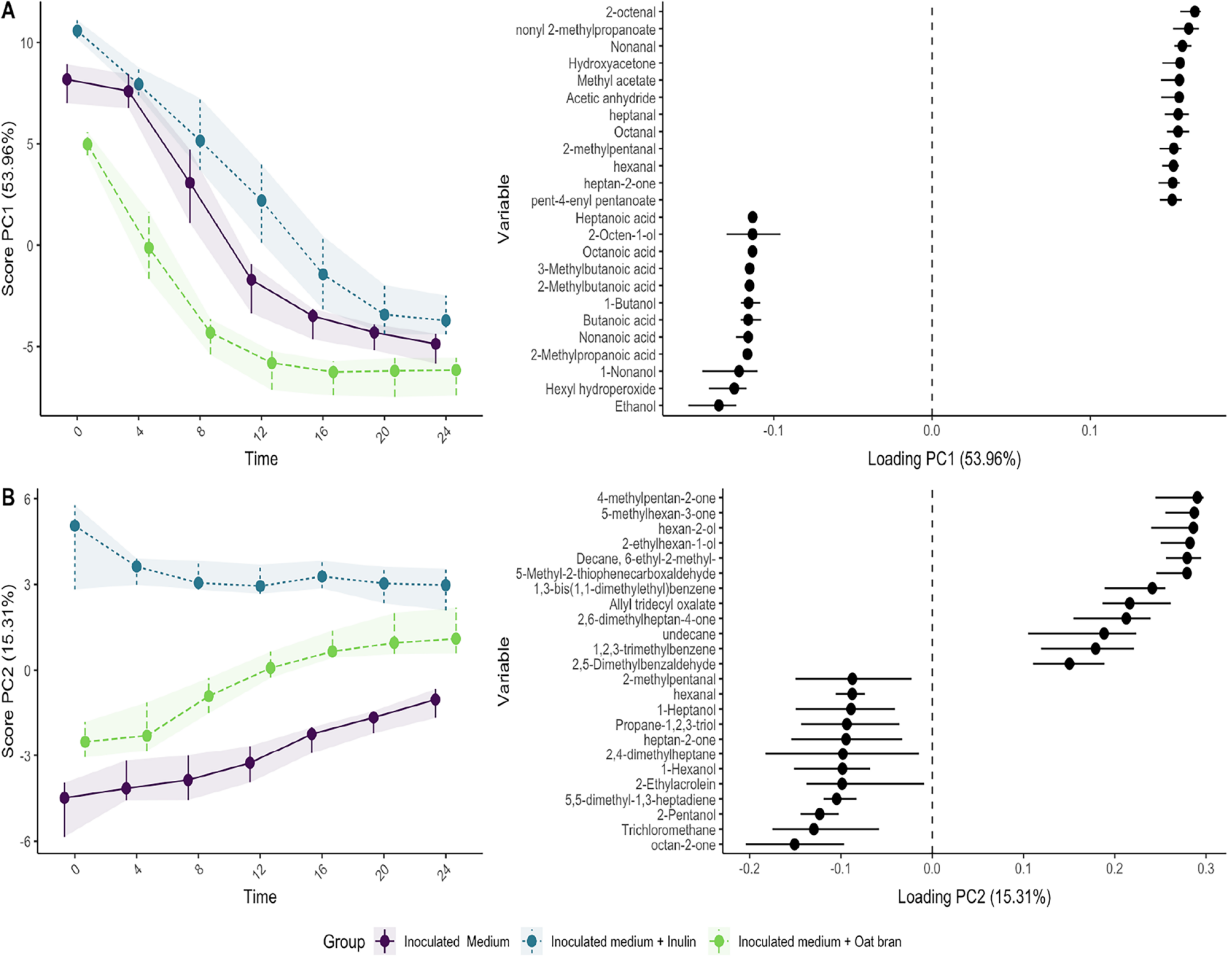
RM-ASCA model explaining the VOCs time development over 24 h of fermentation for three groups: basal medium + inoculum + inulin (blue), basal medium + inoculum + oat bran (green), and basal medium + inoculum (violet). On the left side of both panels scores are shown while loadings on the right. (**A**) and (**B**) indicates principal component 1 and 2 summarising 53.96% and 15.31% of the overall explained variance respectively. Only the VOCs with highest and lowest loadings are shown. The error bars and shaded areas reflect 95% confidence intervals after jack-knife validation n = 1000. The confidence intervals are obtained by jack-knife validation n = 1000.

While the clustermap provided valuable insights into the initial dataset, it has limitations in fully accounting for the influence of experimental factors and repeated measurements on specific volatile compounds. To address these limitations, we applied the repeated measurement ANOVA simultaneous component analysis (RM-ASCA) framework. The methodology combines general linear models with PCA, allowing it to decompose and visualise the separate effects of experimental factors^33^. Figure 2 shows the overall distribution of scores as histograms for principal component 1 (PC1), principal component 2 (PC2) and principal component 3 (PC3). RM-ASCA extracted three main general patterns of change as represented by the PC1, PC2 and PC3 explaining 53.96%, 15.31% and 14.05% of the global variability that characterises the dataset. The evolution in time for the calculated distribution of scores showed how quick changes in VOCs during fermentation are captured by the experimental approach. In particular, Fig. 2 shows how the distribution of scores for PC1 was similar at the starting point for the inoculated saamples, in the situation where the fermentation medium was unaltered, and then gradually changed to a situation where the samples were more distinct in the VOCs profile. The fermentation of different substrates resulted in varying volatilome profiles over time which allowed to distinguish the different experimental conditions. From the analysis, 12 h resulted in the most distinct fermentation profile between the studied groups. In Fig. 3 the representation of the generated RM-ASCA model scores and loadings for PC1 and PC2 is shown.

In Fig. 3, each component was associated with its respective positive and negative loadings. To facilitate interpretation, due to the extensive number of evaluated volatile organic compounds (VOCs), the 12 VOCs that showed the highest and lowest loadings on either side of the vertical dotted line were graphically represented. Negative loadings signified an increase in abundance over time, while positive values indicated trends of consumption. Further elucidation of this pattern was available in Supplementary material (Fig. S3), where individual plots for the most significant molecules were presented.

**Figure 4:**
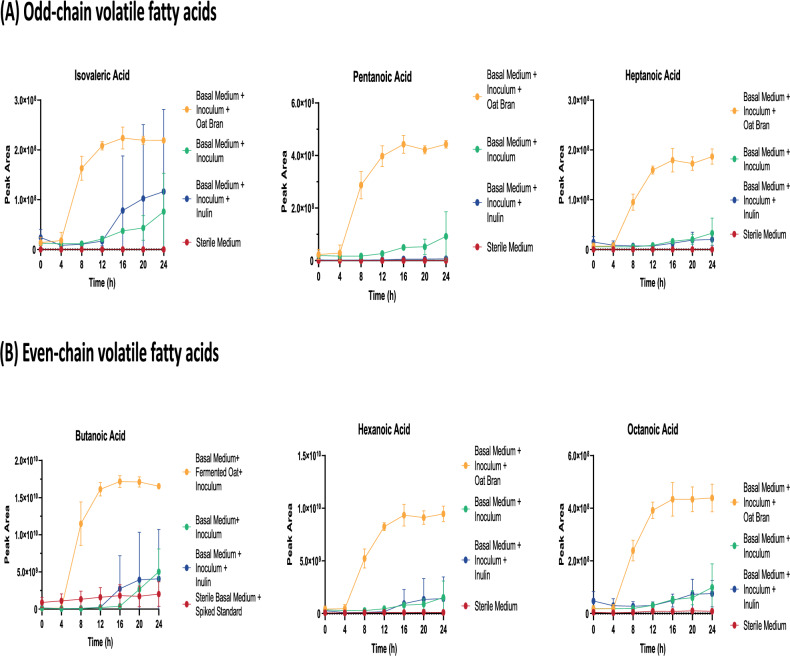
Release kinetics of (**A**) odd-chain and (**B**) even-chain volatile fatty acids during anaerobic in-vitro fermentation. The oat bran fermented sample showed a steep increase of MCFAs between 4 and 8 h of fermentation time. The inulin sample and the inoculated basal medium resulted in the release of MCFAs on a lesser extent. The results suggest a preference for odd-chain acid production during oat bran fermentation. Error bars represent the standard deviation. Data are representative of three independent experiments (n = 3).

By looking at the calculated confidence intervals for each experimental condition, they were non-overlapping, allowing for the discrimination of group-specific VOCs during the fermentation. The combination of RM-ASCA analysis and volatilome longitudinal monitoring allowed to study trends of consumption and production for specific VOCs showing highest and lowest loadings within the dataset. Notably, the group of molecules exhibiting decreasing abundance over time for PC1, included mainly aldehydes (2-octenal, nonanal, heptanal, octanal, 2-methylpentanal, hexanal), and esters (nonyl 2-methylpropanoate, methyl acetate, pent-4-enyl pentanoate) which may indicate initial microbial transformations of these substrates to a different extent, depending on the tested substrates. In contrast, the compounds exhibiting the lowest loadings, showed increasing abundance over time, and were alcohols (2-octen-1-ol, ethanol) and mainly carboxylic acids (heptanoic acid, octanoic acid, 3-methylbutanoic acid, butanoic acid, nonanoic acid). For PC2 ketones (4-methyl-pentan-2-one, 5-methyl-hexan-3-one) and alcohols (2-ethylhexan-1-ol, hexan-2-ol) were the predominant chemical class exhibiting strongest positive loadings. Mainly alcohols (1-heptanol, 1-hexanol, 2-pentanol ) and aldehydes (2-methylpentanal, hexanal, 2,4-dimethylhexanal, 2-ethylacrolein) were associated with negative loadings in PC2. Overall, the fermentation of the basal minimal medium (containing mucin as only fermentable substrate) and inulin, a soluble fibre with knwon prebiotic effect, shared a more similar headspace profile over time compared to oat bran which in mainly insoluble. The volatilome profile of oat bran was more distinct from that of the soluble substrates and was characterised also by the synthesis of several fatty acids. In fact, loadings from PC1 showed an increased abundance over time for fatty acids with different chain lengths in oat bran compared to the basal medium with or without inulin, also in accordance with cluster II previously described in Fig. 1. As illustrated in Fig. 4, the fermentation of the basal medium and the fermentation of inulin still resulted in the release fatty acids, but to a lesser extent and with different kinetics. The kinetic profiles for -odd and -even chain fatty acids revealed increased peak intensities between 4 and 8 h of incubation. A plateau was reached after 16 h fermentation. Figure 4 showed that fermentation of oat bran led to the production of medium-chain fatty acids (MCFAs) with either an even chain length (Fig. 4A) or an odd chain length (Fig. 4B). In order of peak intensities, the released compounds were butanoic acid > hexanoic acid > octanoic acid > pentanoic acid > isovaleric acid > heptanoic acid highlighting a preference towards even-chain compounds.

## Discussion

The proposed GC-MS methodology, that featured non-invasive sampling, automated operations and temperature control, allowed to longitudinally monitor VOCs released during an in-vitro batch system simulating the anaerobic distal colonic fermentation of food matrices (i.e. dietary fibres). It facilitated the assessment 88 VOCs over time in a wide range of chemical classes such as alcohols, aldehydes, esters, ketones, furans, alkanes, thiophenes and medium to and short-chain fatty acids. The potential of this solution is particularly interesting from the perspective of providing a temporal dimension to multi-omics studies targeting in-vitro model systems of the gut microbiota^9^. The non-invasive analysis provided a comprehensive comparison of the overall impact of oat bran, an insoluble dietary substrate resistant to digestion, and a well-known soluble prebiotic, inulin, during a 24-h in-vitro fermentation. Even though both are mainly known to produce short-chain fatty acids as main by-products, their effect on the gut microbiota is also reflected in the overall differences in VOCs produced. In fact, the effect of undigestible dietary substrates in presence of complex anaerobic microbial communities, is often more extended and determines the rapid global change of other metabolites over time, as shown for certain dietary fibres, including inulin^7,34,35^. To fully exploit the longitudinal nature of the acquired data, statistical tools suitable for this approach were employed. The clustering analysis, which organized VOCs into sub-clusters based on similar time-dependent profiles, highlighted the complexity and substrate-specific nature of these microbial transformations starting from the same microbial inoculum. Furtermore, the clustering of VOCs molecular profiles provides useful information about the system’s molecular behaviour^36^. Grouping molecules with the same time pattern is an indication to understand which molecules can be co-regulated or participate in the same biological processes^37^. In this context, the application of RM-ASCA further enhanced the interpretability of the longitudinal dataset, facilitating the elucidation of key VOCs explaining the variability within the studied groups at specific time points. In general, the methodology provides a tool to gain a system-level understanding of anaerobic fermentation processes from individual(s) faecal samples. By looking at the temporal profile of fermentation end-products, which are mainly VOCs, it is possible to hypothesise how gut microbial communities transform undigestible food matrices. As an example, the simultaneous rise in odd- and even-chain carboxylic acids was mainly a characteristic of oat bran fermentation. The release of multiple MCFAs might indicate the concerted action of different microbial groups cooperating in a food breakdown web^38–40^. A similar mechanism where MCFAs were released during the anaerobic breakdown of complex organic matrixes by anaerobic cultures was elucidated using time-resolved metatransciptomics by Scarborough et al.^38^.Furthermore, the release of MCFAs, as an oat bran signature, has been described in few intestinal fermentation experiments in-vitro and recently reported during an in-vivo intervention^36,41^. In general, the possibility to easily monitor and understand the change in VOCs depending on the dietary substrate and donor under study can reveal less-obvious dynamics occurring when studying microbe-diet interactions in-vitro. In a context of personalised dietary interventions, volatilome monitoring could be used to predict overall effects of diet when exposing individual’s faecal samples to multiple substrates, in a similar way we exposed the same inoculum to oat bran or inulin. Furthermore, the possibility to select relevant VOCs profiles also provides a starting point to select pool of molecules to manipulate the gut microbiota in-vitro. The non-invasive cultivation, sampling, and VOC analysis workflow, open scenarios where it is possible to manipulate the system by adding endogenous compounds at specific time points and study the response in terms of released VOCs over time or the change in bacterial composition and functionality. Future studies should aim to extend these findings, potentially linking the production of specific VOCs over time to changes in microbial taxa. About the limitations of the study, the use of a stool sample from a single individual served as a practical starting point for our investigation to test multiple substrates at the same time. Even though we recognize the importance of expanding our sample pool to include multiple individuals to explore inter-individual variability, these results are not meant to be generalized for a certain population. The material used for VOCs sampling (DVB-CAR-PDMS), offers a comprehensive coverage of chemical classes, but has potential limitations associated with adsorption mechanisms^30,42^. In conclusion, this study emphasizes the need to consider the gut microbial headspace not as a static environment but rather as a dynamic chemical space containing information on community behavior when converting dietary substrates. Since VOCs can be used to track biological processes in real-time, the proposed methodology can fill the gap left by static metabolomics approaches in gut microbiota in-vitro research. A dynamic understanding of VOCs may aid in the development of new strategies for modulating the gut microbiota and improving human health.

## Methods

### Fibre sources

Commercial dry-milled oat bran was obtained from a local mill in Wageningen, The Netherlands. The bran-rich fraction was acquired through the debranning process utilizing the Satake Abrasive Test Mill Model TM05C^43^ as described by Kedia et al.^32^. Inulin was purchased from Sigma Aldrich (The, Netherlands)

### Preparation of the faecal stabilized inoculum

The protocol for the preparation of stabilized faecal inoculum was adapted from^44^ with modifications. An adult subject (27 years old), declaring no smoking or antibiotics consumption 6 months prior to the study, donated a fresh faecal sample. The healthy volunteer gave written consent for a single faecal donation, and anonymity was always maintained. According to the guidelines of the Medical Ethical Advisory Committee of Wageningen University (METC-WU), this research did not require ethics approval (METC 2023-16718). The fresh faecal sample was stored in a collection box with an anaerobic AnaeroGen bag (Oxoid, UK) and used within 2 h. A $20\%$ (w/v) solution of the faecal sample was homogenized with phosphate buffer for 10 min using a Stomacher 400 circulator (Seward, UK). The sterilized phosphate buffer consisted of $8.8 \, \text {g} \, \text {L}^{-1} \, \text {K}_2\text {HPO}_4$, $6.8 \, \text {g} \, \text {L}^{-1} \, \text {KH}_2\text {PO}_4$, and $0.1 \, \text {g} \, \text {sodium thioglycolate}$ in demi-water. The pH was adjusted to 7, and $15 \, \text {mg sodium thionite}$ was added. Fresh faecal sample was inoculated in a SHIME system vessel simulating distal colonic conditions to obtain $1\%$ w/v concentration. The vessel was fed using a standard nutritional SHIME medium and was made anaerobic after inoculation by flushing with nitrogen for 10 min every day. The SHIME ran for 10 days according to baseline configurations to establish a stable microbial community. The adaptation time enables the microbial community present in the faeces to re-establish itself in the laboratory bioreactor^45^. After the stabilization period, the microbiota was collected and stored with cryoprotectant (a final concentration of $42\% \, \text {glycerol}$), $0.5 \, \text {g} \, \text {L}^{-1}$ l-cysteine-HCl, $10 \, \text {g} \, \text {L}^{-1} \, \text {trehalose}$, and $3 \, \text {g} \, \text {L}^{-1} \, \text {tryptic soy broth}$ at $-80 \, ^\circ \text {C}$ before use.

### Batch fermentation in anaerobiosis

The batch fermentations were performed directly in 20 mL glass vials with magnetic screw tops and silicone/PTFE septa. Vials were autoclaved, filled with sterile media and substrates of interest (i.e inulin, oat bran) in aseptic conditions. Subsequently, they were flushed with nitrogen for 30 min using sterile needles before inoculation. The confirmation of anaerobiosis through the experiment was ensured by adding resazurin to control bottles. The fermentation, with a final volume of 5 mL, consisted of 3.1 mL of sterile colonic growth medium ($5.22 \, \text {g} \, \text {L}^{-1} \, K_2\text {HPO}_4$, $16.32 \, \text {g} \, \text {L}^{-1} \, KH_2\text {PO}_4$, $2 \, \text {g} \, \text {L}^{-1} \, \text {NaHCO}_3$, $2 \, \text {g} \, \text {L}^{-1} \, \text {yeast extract}$, $2 \, \text {g} \, \text {L}^{-1} \, \text {peptone}$, $1 \, \text {g} \, \text {L}^{-1} \, \text {mucin}$, $0.5 \, \text {g} \, \text {L}^{-1}$ l-cysteine-HCl, $2 \, \text {mL} \, \text {tween-80}$) and $0.5 \, \text {g}$ of substrate dissolved in $1.4 \, \text {mL}$ of sterilized demi-water and $0.5 \, \text {mL}$ of stabilized faecal inoculum to give a final substrate concentration of $1\% (w/v)$ with $10\% (w/v)$ faecal inoculum The inoculum procedure was performed at 37 $^{\circ }$C in a pre-heated anaerobic chamber. The inoculated samples were: basal medium + inulin (I), basal medium + oat bran (O), and basal medium (FM). The noninoculated samples was: Sterile Medium (SM). The full experiment consisted of 3 independent replicates performed using a randomized block design. A graphical representation of the experimental approach is shown in Supplementary material (Fig. S1).

### Headspace solid-phase microextraction

Automated, headspace solid-Phase microextraction (HS-SPME) was used for sampling of VOCs. After inoculation, the vials were incubated at 37 $^{\circ }$C on a heated tray mounted on a triaxial autosampler (CTC Analytics, Zwingen, Switzerland), and exposed for 45 min to a SPME fiber composed of 50/30 μm Divinylbenzene/Carboxen/Polydimethylsiloxan (DVB-CAR-PDMS) (Supelco, PA, USA). The choice of DVB-CAR-PDMS and SPME time was based on previous study by Dixon et al.^42^ and Timm et al.^28^. During the incubation period, the samples were shaken directly on the autosampler using its incubator at the lowest possible speed (250 rpm) to simulate the shaking during faecal batch fermentations. After 45 min exposure of the SPME fiber to the headspace, thermal desorption (5 min) and cooling (10 min) the apparatus was moved to the next sample for new exposure and desorption, every 4 h over the entire incubation time (24 h). The samples were extracted several times over a 24 h period, by non-exhaustive microextraction and not exceeding 37 $^{\circ }$C to maintain their biological integrity. Dynamic VOCs release was monitored at the following time points: 0 h, 4 h, 8 h, 12 h, 16 h, 20 h, 24 h.

### Gas chromatography–mass spectrometry

Analyses were performed in a Clarus500 GC unit (PerkinElmer AutoSystem XL, Waltham, MA, USA) coupled with a mass spectrometer (Clarus500 MS; PerkinElmer, Waltham, MA, USA). The compounds were thermally desorbed from the fiber coating into the GC injector port held at 250 $^{\circ }$C in splitless mode. An HP-Innowax, a polyethylene glycol (PEG) stationary phase fused-silica capillary column (30 m, 0.32 mm ID, 0.5 μm film thickness, J &W Scientific Agilent Technologies, Santa Clara, California, USA) was used to perform the analyte separation. Analyses were performed using helium as the carrier gas at a flow rate of 2 mL $\text {min}^{-1}$. The oven temperature was programmed using a temperature ramp, which started from 40 $^{\circ }$C for 1 min, then increased by 5 $^{\circ }$C $\text {min}^{-1}$ until 250 $^{\circ }$C (2 min). The transfer line temperature was 220 $^{\circ }$C. The mass spectrometer was operated in electron ionization mode (70 eV), with a scan range from m/z 35 to 350. The Kovats indices were calculated by using the retention times of a homologous series of C8-C20 n-alkanes.

### GC-MS data extraction

Using the software PARADISe^46,47^, the GC-MS chromatograms for each type of fermentation were arrayed into a dataset with a three-way structure, X (I J K), where the first mode reflects elution times (I scans), the second the spectral domain (J m/z fragments), and the third samples (K). The chromatographic peaks were chosen as intervals to fit the PARAFAC2 models with non-negativity constraints^46,47^. Maximum fit and core consistency (range 0–100) were used to pick the appropriate number of PARAFAC2 components for each interval. The mass spectrum of each chemical compound was estimated as part of the PARAFAC2 model, which is then used for identification purposes with the NIST MS Search 2.0 software. Each mass spectrum obtained by PARAFAC2 was identified by comparing it to the spectra in the NIST 14 database^48^. To further confirm the identification the calculated Kovats retention indexes were compared to the indexes described in the literature.

### Graphical representations and multivariate statistics

The clustermap was created with Seaborn^49^, applying the Euclidean distance and Ward method for hierarchical clustering in Python. Concentration-time curves were generated with GraphPad Prism 8.0 (GraphPad Software, Inc., San Diego, CA). Before statistical analyses data filtering and scaling were performed. The purpose of the data filtering was to identify and remove variables that are unlikely to be of use when modelling the data. We filtered noninformative variables that were near-constant throughout the experiment conditions by calculating the variation coefficient and filtering out the molecules with low variation coefficient^37^. From the ALASCA package in R^33^, a regression model with a time effect, a time-group interaction, and a random intercept was defined. Time and group (included in the model as ’time:group’) include the interaction between the first time point (i.e., baseline) and group, which has to be removed. Scaling using the built-in function was performed before building the model. The model was built to analyse the combined effect of time and group for the designed experiment. The nonparametric bootstrapping method is employed for validation, resulting in 95% confidence intervals for the scores and loadings associated with each effect matrix. Model validation was performed with jack-knife procedure (n = 1000)^33^.

### Supplementary Information

Supplementary Information 1.

Supplementary Information 2.

Supplementary Information 3.

Supplementary Information 4.

Supplementary Figures.

## Data Availability

The data and the code to perform the analyses are available as supplementary information online and at the Zenodo repository created for this study at the following link: https://doi.org/10.5281/zenodo.10805018.
