# Supplementary material for: Non-invasive VOCs detection to monitor the gut microbiota metabolism in-vitro
Source: Sci Rep. 2024 Jul 9;14:15842. doi: 10.1038/s41598-024-66303-7 (PMC11233675; doi:10.1038/s41598-024-66303-7)
Supplement: Supplementary file 2 — Supplementary Information 2. [file 41598_2024_66303_MOESM2_ESM.pdf]

In [ ]:

```python
import matplotlib.pyplot as plt
import seaborn as sbn
import pandas as pd
from matplotlib.patches import Patch

# read data from an excel file
df = pd.read_excel('report_heatmap.xlsx')

# group data by Sample_time and calculate the mean of each group
df_mean = df.groupby(["Sample_time"]).mean()

# transpose the data to have the heatmap in the right format
df_mean_t = df_mean.T

# create a palette of colors for the different times in the dataset
my_palette = dict(zip(df_mean.Time.unique(), sbn.color_palette("YlOrBr", 10)))

# create row colors based on the time values in the dataset
row_colors = df_mean.Time.map(my_palette)

# set font scale to make the font larger
sbn.set(font_scale=2.8)

# sort the time values and round them to integers
df_mean_time_r = df_mean.Time.sort_values().round().astype(int)

# create a legend with the different time values and their corresponding colors
lut = dict(zip(df_mean_time_r.unique(), row_colors))
handles = [Patch(facecolor=lut[name]) for name in lut]
#plt.legend(handles, lut, title='Time_hours',
#           bbox_to_anchor=(1, 1), bbox_transform=plt.gcf().transFigure, loc='upper right')

# remove the time and other columns from the data that we don't want to plot
df_mean_heat_t = df_mean_t.drop(["Time"], axis=0)

# create a clustered heatmap of the data
sbn.clustermap(df_mean_heat_t, metric="euclidean", cmap="viridis", method="ward", z_score=0,center=0, annot=False, figsize=(70, 70), col_colors=df_mean.Time.map(lut), a

# add legend to the plot
plt.legend(handles, lut, title='Time (hours)',
           bbox_to_anchor=(1, 1), bbox_transform=plt.gcf().transFigure, loc='upper right')
```
