# Supplementary material for: Non-invasive VOCs detection to monitor the gut microbiota metabolism in-vitro
Source: Sci Rep. 2024 Jul 9;14:15842. doi: 10.1038/s41598-024-66303-7 (PMC11233675; doi:10.1038/s41598-024-66303-7)
Supplement: Supplementary file 4 — Supplementary Information 4. [file 41598_2024_66303_MOESM4_ESM.pdf]

In [ ]:

```
---
title: "RM-ASCA ON LONGITUDINAL VOCS DATASET DURING IN-VITRO BATCH FERMENTATION"
author: "Dell'Olio A."
date: "2024-03-11"
output: pdf_document
---

```{r setup, include=FALSE, echo=FALSE}
#Data pre-treatment
#Loading libraries
library(tinytex)
library(readxl)
library(tidyr)
library(dplyr)

# Read the data
GCMSData_ALASCA_nm <- read_excel("~/Desktop/GCMS_alasca_nomedium.xls")

# Define function to filter out features with low coefficient of variation
remove.low.cv <- function(X, cutoff = 0.5){
  cv <- unlist(lapply(as.data.frame(X),
                      function(x) abs(sd(x)/mean(x))))
  return(X[,cv > cutoff])
}

# Fill zeros using mean within column
GCMSData_ALASCA_nm_filled <- GCMSData_ALASCA_nm %>%
  mutate(across(4:91, ~ifelse(. == 0, mean(., na.rm = TRUE), .)))

# Subset dataset removing metadata
GCMSData_ALASCA_nm_filled_sub <- GCMSData_ALASCA_nm_filled[, 4:91]

# Filter out features with low coefficient of variation
GCMSData_ALASCA_nm_filled_sub_filtered <- remove.low.cv(GCMSData_ALASCA_nm_filled_sub, 0.5)

# Isolate metadata and data and re-bind the whole
metadata <- GCMSData_ALASCA_nm_filled[, 1:3]
Filtered_GCMS_ALASCA <- cbind(metadata, GCMSData_ALASCA_nm_filled_sub_filtered)

# Transform the dataset in the long form
GCMSData_ALASCA_Long <- gather(Filtered_GCMS_ALASCA, variable, value, 4:73) %>%
  select(ID, time, group, variable, value) %>%
  arrange(time)

# Convert 'time' to a factor
GCMSData_ALASCA_Long_nm <- mutate(GCMSData_ALASCA_Long, time = as.factor(time))

# Perform RM-ASCA
library(ALASCA)

Alasca_GCMS_model_nm <- ALASCA(
  GCMSData_ALASCA_Long_nm,
  formula = value ~ time:group + (1|ID),
  separate_effects = FALSE,
  scale_function = 'sdall',
  reduce_dimensions = FALSE,
  validate = TRUE,
  n_validation_runs = 1000,
  validation_method = 'jack-knife'
)

# Plot data with built-in function
plot(Alasca_GCMS_model_nm, effect = 1, component = c(1,2,3), type = 'effect')
plot(Alasca_GCMS_model_nm, effect = 1, component = c(1,2), type = 'validation')
plot(Alasca_GCMS_model_nm, effect = 1, component = c(2), type = 'prediction')
plot(Alasca_GCMS_model_nm, effect = 1, component = c(1), type = 'participants')
plot(Alasca_GCMS_model_nm, effect = 1, component = c(2), type = 'participants')
plot(Alasca_GCMS_model_nm, effect = 1, component = c(3), type = 'participants')
histo_pc1 <- plot(Alasca_GCMS_model_nm, effect = 1, component = c(1), type = 'histogram')
histo_pc2 <- plot(Alasca_GCMS_model_nm, effect = 1, component = c(2), type = 'histogram')
histo_pc3 <- plot(Alasca_GCMS_model_nm, effect = 1, component = c(3), type = 'histogram')
```
```
