# Supplementary material for: Non-invasive VOCs detection to monitor the gut microbiota metabolism in-vitro
Source: Sci Rep. 2024 Jul 9;14:15842. doi: 10.1038/s41598-024-66303-7 (PMC11233675; doi:10.1038/s41598-024-66303-7)
Supplement: Supplementary file 5 — Supplementary Figures. [file 41598_2024_66303_MOESM5_ESM.pdf]

Non-invasive VOCs detection to monitor the gut microbiota metabolism *in-vitro*: supplementary material

1. SUPPLEMENTARY FIGURES

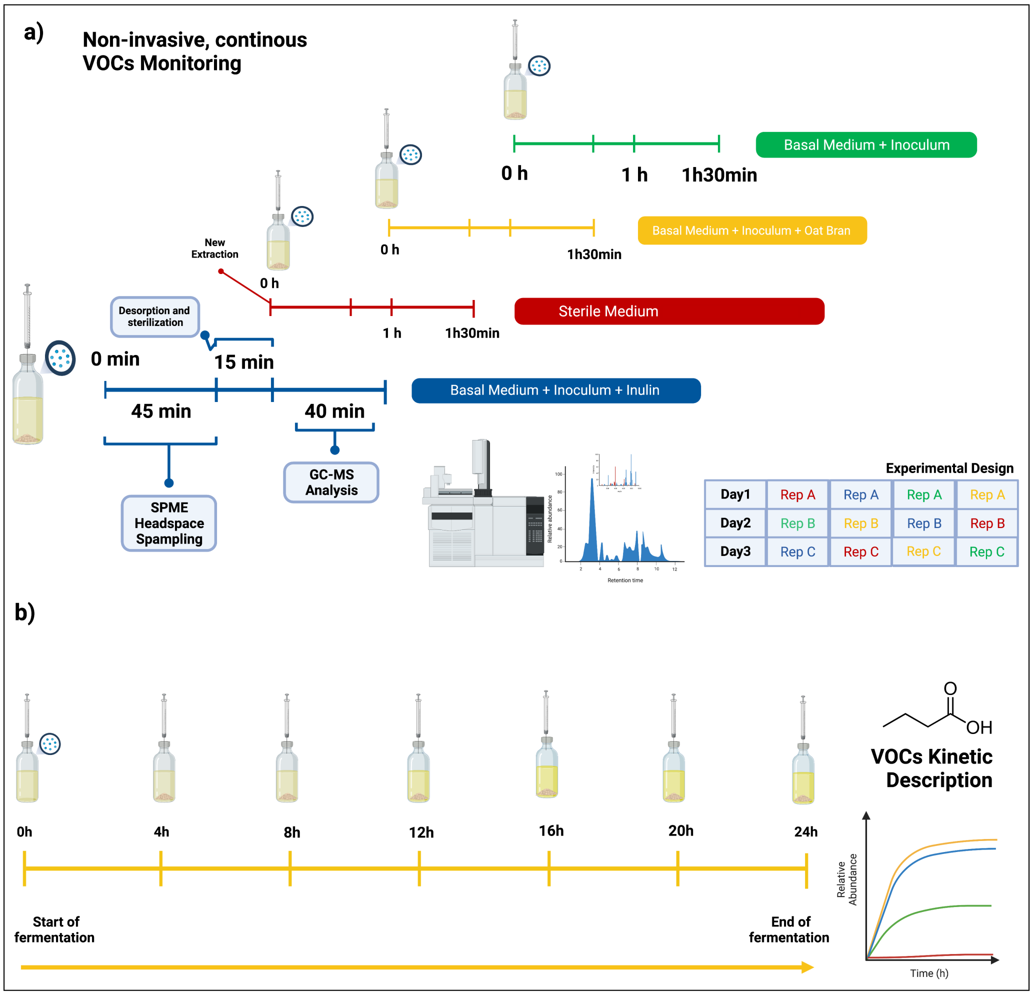

Fig. S1. a) A graphical representation of the experimental Design. The full experiment which consisted of 3 independent replicas of 4 sample type, were performed using a randomized block design with the day of experiment as blocking factor. The inoculated samples were: basal medium + inulin, basal medium + oat bran, and basal medium. The non-inoculated samples were: Sterile Medium (SM) (b) The term "non-invasive" in our study refers mainly to the experimental procedure adopted to preserve the integrity of the biological sample. In detail, the repeated extraction of the same sample set over a 24 hour period, without exceeding the extraction temperature of 37°C, preserves the integrity of the biological sample during the analysis. Furthermore, is also related to the non-exhaustive microextraction procedure, which removes only a small portion of the target analytes from the matrix. The procedure causes minimum perturbation to the microbial headspace compared more invasive approaches.

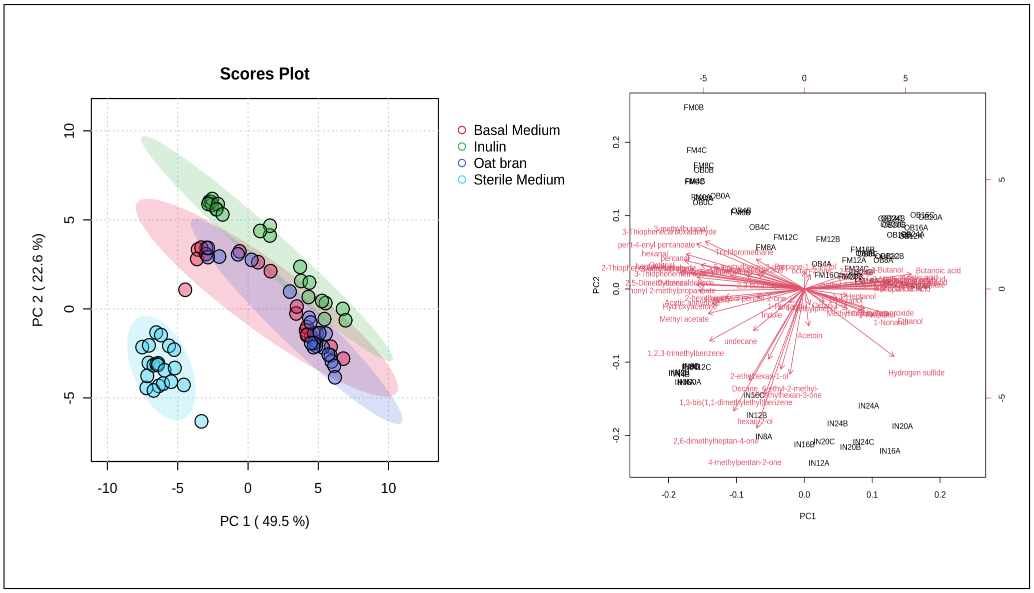

Fig. S2. On the right Principal Component Analysis (PCA) showing the explained variance for PC1 and PC2 and the distribution between sample types. Sterile media sample data were found in a dense cluster when compared to fermented samples. On the right PCA biplot showing the relationship between sample types and the released metabolites. The Labels are basal medium + inulin (IN), basal medium + oat bran (OB), and basal medium (FM). The replicas are indicated by A,B and C. The Numeric part of the label indicates the time points. Log10 transformation and pareto scaling was performed before PCA.

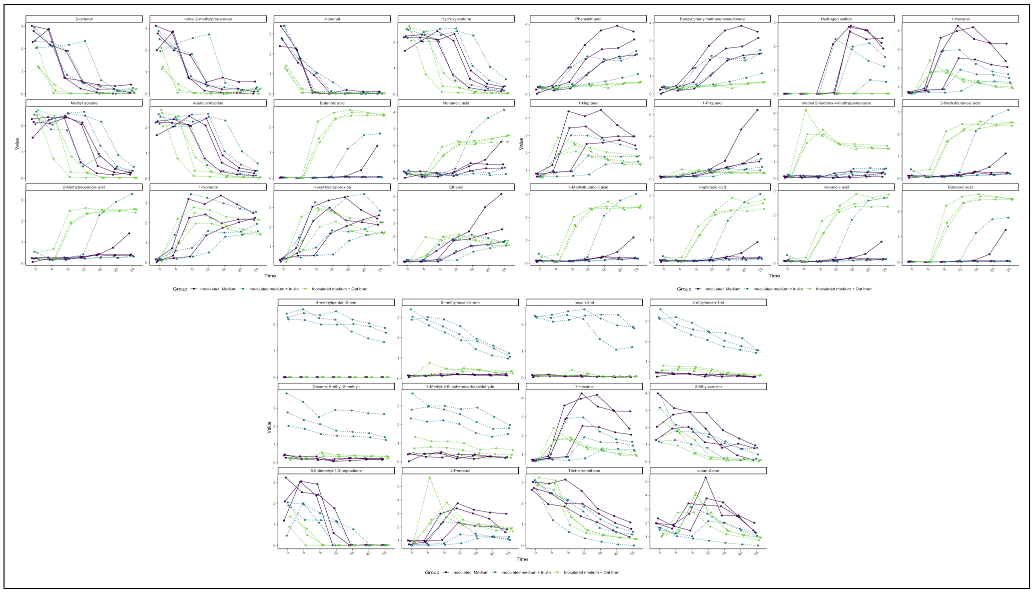

Fig. S3. This figure shows individual abundance-time curves of compounds selected by RM-ASCA. The curves were obtained by coupling automated HS-SPME-GC-MS to anaerobic *in-vitro* batch fermentation system. The curves are representative of three independent experiments. (n=3)
